# Supplementary material for: Magnetic hyperbolic optical metamaterials
Source: Nat Commun. 2016 Apr 13;7:11329. doi: 10.1038/ncomms11329 (PMC4833869; doi:10.1038/ncomms11329)
Supplement: Supplementary Information — Supplementary Figures 1-6, Supplementary Notes 1-7 and Supplementary References. [file ncomms11329-s1.pdf]

SUPPLEMENTARY FIGURES

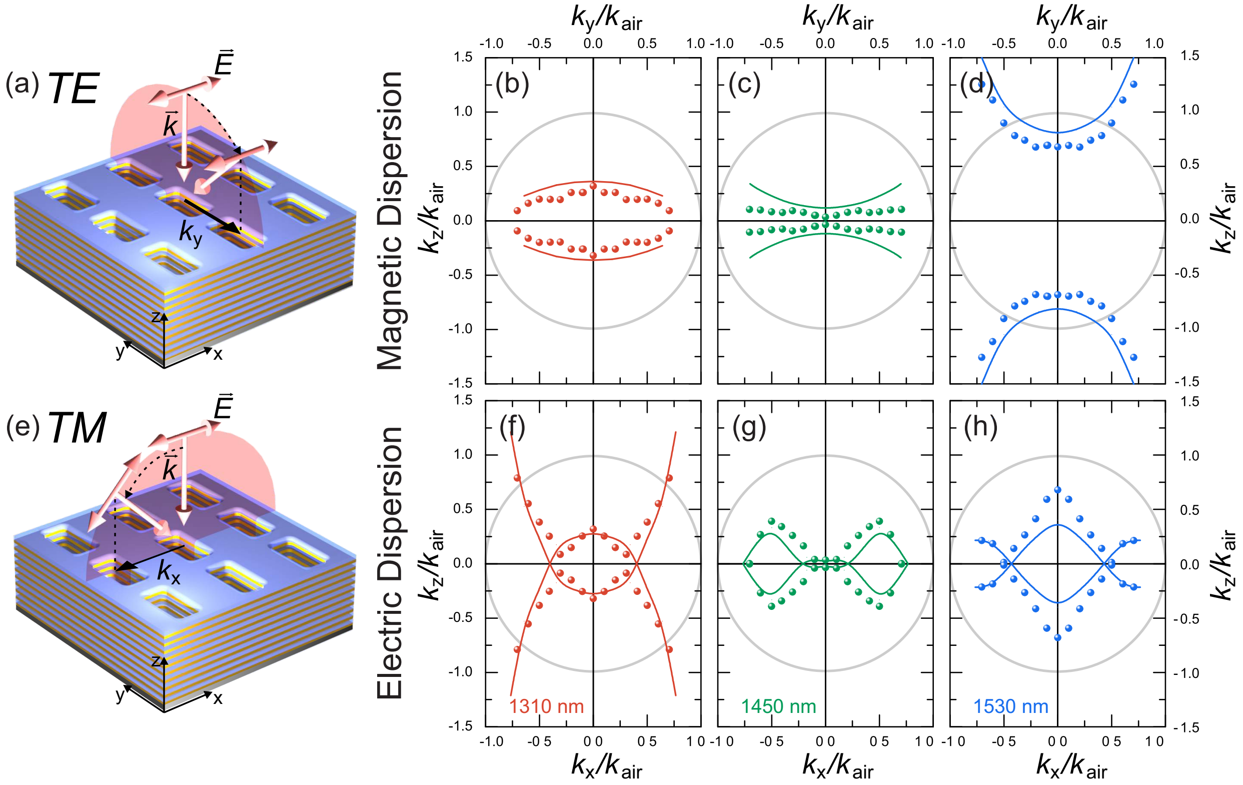

Supplementary Figure 1. Comparison of experimental results with numerical calculations. (a) Sketch of TE-illumination geometry. (b-d) Isofrequency dispersion contours for TE-polarization at wavelengths 1310 nm, 1450 nm, and 1530 nm, respectively. (e) Sketch of TM-illumination geometry. (f-h) Isofrequency dispersion contours for TM-polarization at wavelengths 1310 nm, 1450 nm, and 1530 nm, respectively. Dots mark the experimental data, and lines correspond to numerical results. Grey circles correspond to an isofrequency contour of light in vacuum.

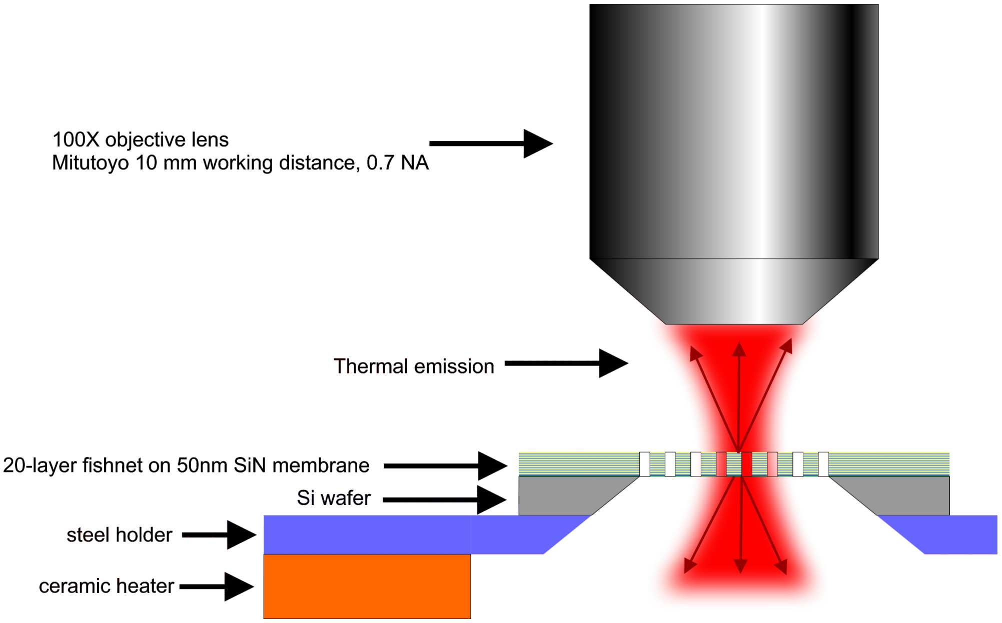

Supplementary Figure 2. Schematic of sample heating and collection of thermal radiation

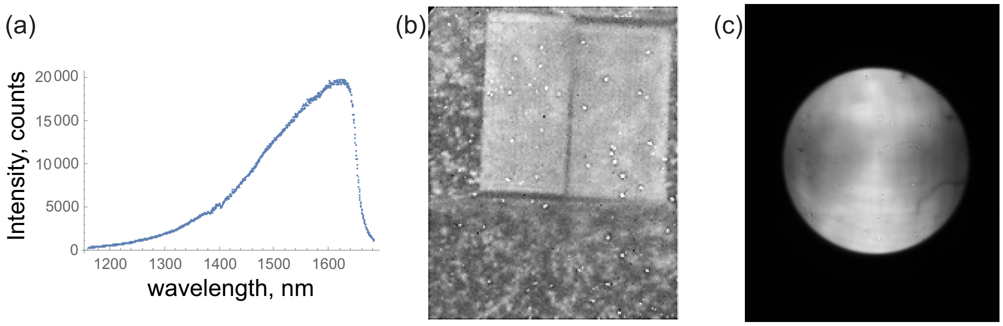

Supplementary Figure 3. Raw data for thermal radiation (a) spectra, (b) real space raw image of thermal emission from 50X50 μm fishnet sample and surrouded unpatterned multilayer structure (full spectrum), (c) back focal plane raw image of thermal emission from a fishnet sample (full spectrum)

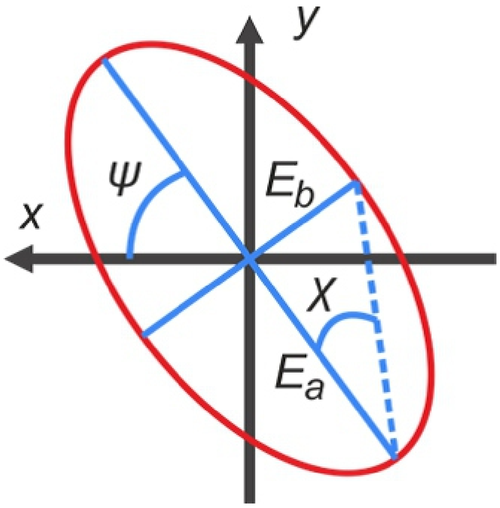

Supplementary Figure 4. Schematic of the polarization ellipse with the Stokes coefficients of ellipticity angle χ and polarization-inclination angle ψ. E_a and E_b are the main polarization axes (solid blue lines) of the polarization ellipse.

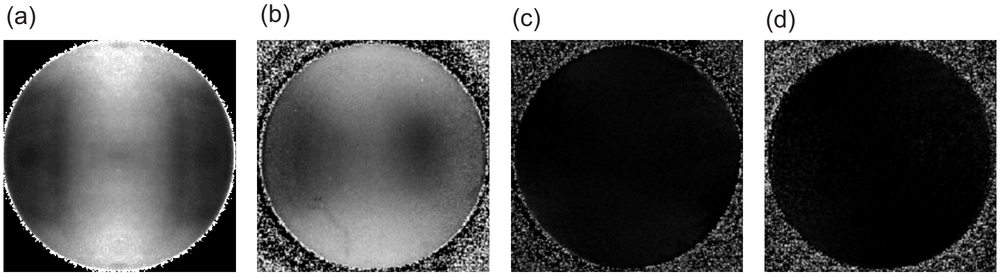

Supplementary Figure 5. Set of Stokes coefficients: (a) intensity, (b) polarization degree, (c) polarization inclination, (d) ellipticity.

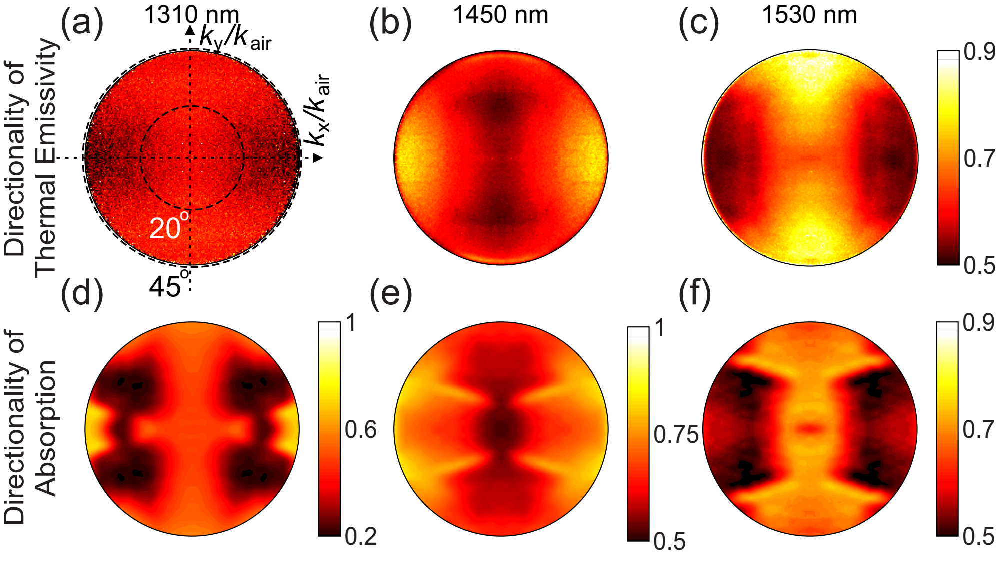

Supplementary Figure 6. (a-c) Directionality of thermal emissivity at 400°C at wavelengths 1310 nm, 1450 nm, and 1530 nm, respectively. (d-f) Directionality of absorption at room temperature for the same three wavelengths 1310 nm, 1450 nm, and 1530 nm, respectively.

SUPPLEMENTARY NOTES

Supplementary Note 1

Magnetic hyperbolic dispersion. Light is an electromagnetic wave, *i.e.* harmonic oscillations of electric and magnetic fields periodic in time and in space. Periodicity of light wave in time is defined by its frequency ω. And its periodicity in space is defined by the wave-vector, or $\mathbf{k}$-vector. Time and space periodicity of light are connected together with the dispersion relation. Importantly, the dispersion is largely defined by properties of a given optical medium. We will first consider a special case of *local* media. This assumes that the electric displacement vector $\mathbf{D}$ and the magnetic field $\mathbf{H}$ at a given point in space can be written in terms of averaged electric fields $\mathbf{E}$ and average induction field $\mathbf{B}$. For the local media the electromagnetic properties are defined by the tensors of electric permittivity ε and magnetic permeability μ

$$\hat{\epsilon} = \begin{pmatrix} \epsilon_x & 0 & 0 \\ 0 & \epsilon_y & 0 \\ 0 & 0 & \epsilon_z \end{pmatrix}; \qquad \hat{\mu} = \begin{pmatrix} \mu_x & 0 & 0 \\ 0 & \mu_y & 0 \\ 0 & 0 & \mu_z \end{pmatrix}. \tag{1}$$

With this definition of the tensors we leave out of consideration media exhibiting gyrotropy or magnetoelectric coupling.

For a given frequency ω all the $\mathbf{k}$-vectors belong to a certain three-dimensional surface, called *isofrequency surface*. And the shape of the isofrequency surface depends on the material parameters $\hat{\varepsilon}$ and $\hat{\mu}$. To analyze the possible shapes of these surfaces we write explicitly a set of two equations for two principal linear polarizations: TE and TM. Without lack of generality we assume that for TE-polarization electric component of wave is pointing in x-direction, and for TM-polarization magnetic component is pointing in y-direction. The resulting dispersion relations take form:

$$\text{TE:} \quad \frac{k_y^2}{\varepsilon_x \mu_z} + \frac{k_z^2}{\varepsilon_x \mu_y} = \frac{\omega^2}{c^2}; \qquad \text{TM:} \quad \frac{k_x^2}{\varepsilon_z \mu_y} + \frac{k_z^2}{\varepsilon_x \mu_y} = \frac{\omega^2}{c^2}. \tag{2}$$

These equations describe the two types of isofrequency contours: either *elliptic* or *hyperbolic* depending on the relative signs of the components of $\hat{\varepsilon}$ and $\hat{\mu}$ tensors. Essentially, in the media with hyperbolic dispersion, the tensors $\hat{\varepsilon}$ or $\hat{\mu}$ have diagonal components of

opposite signs. If the tensor of electric permittivity $\hat{\varepsilon}$ has both positive and negative components, this results in hyperbolic dispersion for the TM-polarization, or *electric* hyperbolic dispersion. Similarly, components with opposite signs in magnetic permeability μ tensor result in hyperbolic dispersion for the TE-polarization, or *magnetic* hyperbolic dispersion.

Supplementary Note 2

Determination of k-vector from complex transmission and reflection coefficients. The normal component of the vector k_z can be found as [1–5]:

$$k_z = \pm\frac{1}{h}\cos^{-1}\left(\frac{1-r^2+t^2}{2t}\right)+\frac{2\pi m}{h}, \tag{3}$$

where t and r are complex transmittance and reflectance, h is the thickness of the material slab and m is an integer number. Complex transmission and reflection coefficients carry the information about both the amplitude and the phase of light. The two tangential components k_x and k_y remain continuous at the interface between the media according to the boundary conditions.

Supplementary Note 3

Phase retrieval technique. We experimentally detect an interference pattern of the sample wave and reference wave coming at an angle with respect to each other. We perform a Fourier-transform of the image of the interference pattern and in Fourier-image we filter-out all the spatial frequencies except the frequencies corresponding to a single maximum of the first order. After the filtering, we perform the inverse Fourier-transform, which gives us a two-dimensional distribution (image) of a *complex* field, where the phase of the field represents a phase difference between the sample and reference beams. We normalize our transmission measurements (both amplitude and phase) to the transmission through an empty space, and normalize the reflection measurements to the reflection from a golden mirror.

Supplementary Note 4

Spatial dispersion. In some cases approximation of local medium is not sufficient to describe the optical properties of media and corresponding dispersion. In this case the theory of *spatial dispersion* needs to be employed [6, 7]. Within this theory components of electric permittivity tensor of the medium are considered to be a function of a k-vector $\varepsilon = \varepsilon(\mathbf{k})$. This can be expanded into Taylor series by $\mathbf{k}$. Here we will consider centra-symmetric media. In such media even terms of Taylor expansion vanish. Thus, the expression for ε in the case of spatial dispersion takes form:

$$\varepsilon(\mathbf{k}) = \varepsilon^{loc} + \Sigma_{ij}\frac{\partial^2\varepsilon}{\partial k_i \partial k_j}k_i k_j + \dots . \tag{4}$$

The tensor $\Sigma_{ij}\frac{\partial^2\varepsilon}{\partial k_i \partial k_j}$ is also called quadrupole susceptibility tensor. We further take the quadrupole susceptibility into consideration and neglect higher-order terms. The quadrupole tensor may have up to six independent components in non-symmetric structures, while symmetries reduce this number [8, 9].

We further focus on spatial dispersion of multilayer fishnet metamaterials. We assume our electric field to be in the $x - z$ plane, thus we consider ε_x and ε_z components of the electric permittivity tensor.

Our dispersion relation for TM-polarization take form:

$$\text{TM:} \quad \frac{\omega^2}{c^2} = \frac{k_x^2}{\varepsilon_z(\mathbf{k})} + \frac{k_z^2}{\varepsilon_x(\mathbf{k})} = \frac{k_x^2}{\varepsilon_z^{loc} + \frac{\partial^2\varepsilon_z}{\partial k_x^2}k_x^2 + \frac{\partial^2\varepsilon_z}{\partial k_z^2}k_z^2} + \frac{k_z^2}{\varepsilon_x^{loc} + \frac{\partial^2\varepsilon_x}{\partial k_x^2}k_x^2 + \frac{\partial^2\varepsilon_x}{\partial k_z^2}k_z^2}. \tag{5}$$

Here we take into account that quadrupole susceptibility cross-components $\frac{\partial^2\varepsilon_i}{\partial k_x \partial k_z} = \frac{\partial^2\varepsilon_i}{\partial k_z \partial k_x} = 0$ due to the C_2 point symmetry of the structure. The dispersion isofrequency contours are correspondingly the fourth-order curves.

For the case of TE-polarization the only non-zero component of quadrupole susceptibility is $\frac{\partial^2\varepsilon_x}{\partial k_z^2}$, and the other components vanish due to symmetry.

Importantly, this component can be expressed via *local* magnetic permeability $\frac{\omega^2}{c^2}\frac{\partial^2\varepsilon_x}{\partial k_z^2} = 1 - \frac{1}{\mu_y}$ [9, 10]. Thus local and non-local descriptions are *equivalent* for the case of TE-polarization in fishnets. The corresponding dispersion relation in the non-local description takes form:

$$\text{TE:}\quad \frac{k_y^2}{\varepsilon_x^{loc}} + \frac{k_z^2}{\varepsilon_x^{loc}\left(\frac{1}{1-\frac{\omega^2}{c^2}\frac{\partial^2\varepsilon_x}{\partial k_z^2}}\right)} = \frac{\omega^2}{c^2}; \tag{6}$$

And the dispersion isofrequency contours are the second-order curves (such as ellipses or hyperbolas).

To sum up, the isofrequency dispersion of fishnet metamaterials for TE-polarization can be described by *two independent parameters*: either (ε_x^{loc}, μ_y^{loc}), or (ε_x^{loc}, $\frac{\partial^2\varepsilon_x}{\partial k_z^2}$). For TM-polarization the total number of parameters is six. However we note, that two of them are same as for TE-polarization. Therefore, the dispersion for TM-polarization can be described by extra *four independent parameters*: (ε_z^{loc}, $\frac{\partial^2\varepsilon_x}{\partial k_x^2}$, $\frac{\partial^2\varepsilon_z}{\partial k_x^2}$, $\frac{\partial^2\varepsilon_z}{\partial k_z^2}$).

Supplementary Note 5

Numerical simulations of complex dispersion. In addition, we perform numerical calculations of the dispersion of the metamaterial using CST Microwave Studio commercial software. In the calculations we use realistic material parameters of gold, magnesium fluoride and silicon nitride [11–14]. Theoretically calculated and experimentally measured isofrequency contours are in a good agreement with each other.

Supplementary Note 6

Thermal emission experiments. The fishnet sample was heated up to 400°C with custom-built ceramic heating element. Schematic of the heater is shown below. The thermal emission is collected with a 0.7NA and 10mm WD objective lens. Design of the sample holder and the heater was optimized to ensure that thermal emission from the heater and holder is not captured by the objective lens, i.e. only emission from the fishnet is detected. In order to minimize thermal stress during heating/cooling, the temperature of the sample was changed slowly (on the order of hours). For this a custom temperature controller was developed based on Arduino microcontroller.

Thermal emission spectra were measured with Princeton Instruments spectrometer and Andor camera with Peltier-cooled detector (-60°C operation temperature). To calculate the emissivity of the fishnets, the emission spectra of the sample were normalized to emission

spectra of Si at the same temperature. Real space images and back-focal plane images of thermal radiation of fishnets were taken by an IR camera (Xenics, Peltier-cooled, -50°C operation temperature). For making thermal images at specific wavelengths Thorlabs band-pass filter were used with 10 nm FWHM. To resolve polarization states of the emission a quarter-wave plate and a polarizer were inserted into the optical path. The polarization states of both the spectra and the directionality diagrams were retrieved by measuring the full Stokes vectors of the emission. In figure 3 we provide examples of measured thermal spectra and images.

The Stokes coefficeints provide a complete description of the polarization state of light in terms of its total intensity I_{tot}, (fractional) degree of polarization ρ, polarization inclination angle ψ, and the ellipticity angle χ. The ellipticity $\tan(\chi)$ is defined as the ratio of the two axes of the polarization ellipse (see Fig. 4), and the polarization inclination is described by the angle between the main polarization axis and the x-axis of the laboratory coordinate system.

Experimentally, we find the Stokes parameters by measuring the total intensity $I(\theta, \phi)$ where θ is the angle between the direction of the linear polarizer's axis and the y-axis of our laboratory coordinate system. ϕ is the extra phase delay between the two orthogonal linear components of the electric field of the incident wave, that is introduced by the quarter-wave plate. The measured Stokes parameters in the back-focal plane are then given by

$$S_0 = I(0^o, 0) + I(90^o, 0) = I_{tot} \tag{7}$$

$$S_1 = I(0^o, 0) - I(90^o, 0) = \rho I_{tot} \cos 2\psi \cos 2\chi \tag{8}$$

$$S_2 = I(45^o, 0) - I(135^o, 0) = \rho I_{tot} \sin 2\psi \cos 2\chi \tag{9}$$

$$S_3 = I(45^o, \frac{\pi}{2}) - I(135^o, \frac{\pi}{2}) = \rho I_{tot} \sin 2\chi \tag{10}$$

In figure 5 we examplary show a set of Stokes coefficients for the thermal radiation of the fishnet sample at 1530 nm wavelength..

In addition, we compare the far-field thermal emission directionality with the directionality of absorption of the sample [see Figs. 6] calculated as $1 - T - R$, where T and R are transmission and reflection directionalities. The second law of thermodynamics requires absorption and thermal emission to have the same pattern. We observe that, while the resolution of directionality diagrams for thermal emission is lower due to increased material loss

with the increase of the temperature as well as experimental limitations, the diagrams share the same patterns. The fact that in the magnetic hyperbolic regime the thermal emission is *directional* implies that the emission is *spatially coherent.*

Supplementary Note 7

Numerical calculations of thermal emission. We provide full-wave numerical simulations of the thermal emission directionality and spectra. We rely on the fact that absorption equals emission, and therefore we find emission by calculating absorption. In our calculations we take into account change of resistivity of gold with temperature by approx. 2.6 times [15]. Our numerical simulations show remarkable agreement with experiment for polarized and directional fraction of the emission, however the simulations show smaller portion of unpolarized non-directional emission (thermal background). In experiment we attribute higher thermal background to fabrication imperfections such as implantation of ions into the material during the focused ion beam milling and slight deviations of the geometrical parameters. We take this fact into account by introducing a flat non-structured thermal background of constant emissivity 0.3 to the calculated spectra and directionalities. Theoretical spectrum shows the emission averaged over directions within a 45 degree cone. This resembles experimental condition of emission collection with 0.7 NA objective lens.

SUPPLEMENTARY REFERENCES

[1] D. Smith, S. Schultz, P. Markoš, and C. Soukoulis, Phys. Rev. B **65**, 195104 (2002).

[2] C. Menzel, C. Rockstuhl, T. Paul, F. Lederer, and T. Pertsch, Phys. Rev. B **77**, 195328 (2008).

[3] C. Fietz, Y. Urzhumov, and G. Shvets, Opt. Express **19**, 19027 (2011).

[4] S. S. Kruk, D. A. Powell, A. Minovich, D. N. Neshev, and Y. S. Kivshar, Opt. Express **20**, 15100 (2012).

[5] G. T. Papadakis, P. Yeh, and H. A. Atwater, Phys. Rev. B **91**, 155406 (2015).

[6] M. A. Shapiro, G. Shvets, J. R. Sirigiri, and R. J. Temkin, Opt. Lett. **31**, 2051 (2006).

[7] A. A. Orlov, P. M. Voroshilov, P. A. Belov, and Y. S. Kivshar, Phys. Rev. B **84**, 045424 (2011).

[8] H. F. Jones, *Groups, representations and physics* (CRC Press, 1998).

[9] M. A. Gorlach and P. A. Belov, Phys. Rev. B **92**, 085107 (2015).

[10] M. G. Silveirinha, Phys. Rev. B **75**, 115104 (2007).

[11] H. R. Philipp, J. Electrochem. Soc. **120**, 295 (1973).

[12] J. Kischkat, S. Peters, B. Gruska, M. Semtsiv, M. Chashnikova, M. Klinkmüller, *et al.*, Appl. Opt. **51**, 6789 (2012).

[13] H. Li, Journal of Physical and Chemical Reference Data **9**, 161 (1980).

[14] P. B. Johnson and R.-W. Christy, Phys. Rev. B **6**, 4370 (1972).

[15] D. R. Lide, *Handbook of Chemistry and Physics*, 75th ed., 11-41 (New York: CRC Press, 1996-1997).
